# Fatty Acid Profiles of In Vitro Digested Processed Milk

**DOI:** 10.3390/foods6110099

**Published:** 2017-11-09

**Authors:** Michael H. Tunick, Diane L. Van Hekken

**Affiliations:** 1 Center for Food and Hospitality Management, Drexel University, 101 North 33rd Street, Philadelphia, PA 19104, USA; mht39@drexel.edu; 2 Dairy and Functional Foods Research Unit, Eastern Regional Research Center, Agricultural Research Service, United States Department of Agriculture (USDA), 600 East Mermaid Lane, Wyndmoor, PA 19038, USA

**Keywords:** milk, fatty acids, digestion, pasteurization, homogenization, omega-3 fatty acids, conjugated linoleic acid

## Abstract

Digestion of milkfat releases some long-chain (18-carbon) fatty acids (FAs) that can provide health benefits to the consumer, yet because they are found in small amounts and can be difficult to identify, there is limited information on the effects that common fluid milk processing may have on the digestibility of these FAs. This study provides FA profiles for raw and combinations of homogenized and/or heat-treated (high and ultra-high temperature pasteurization) milk, before and after in vitro digestion, in order to determine the effects of processing on the digestibility of these healthy fatty acids. Use of a highly sensitive separation column resulted in improved FA profiles that showed that, when milk was subjected to both pasteurization and homogenization, the release of the 18-carbon FAs, oleic acid, linoleic acid (an omega-6 FA), rumenic acid (a conjugated linoleic acid, CLA), and linolenic acid (an omega-3 FA) tended to be higher than with either pasteurization or homogenization, or with no treatment. Milk is noted for containing the omega-3 FAs and CLAs, which are associated with positive health benefits. Determining how processing factors may impact the components in milk will aid in understanding the release of healthy FAs when milk and dairy foods are consumed.

## 1. Introduction

Milk is an important part of the diet of many people. Digestion of milk fat, 98% in the form of triacylglycerides (TAG), releases bioactive fatty acids that are absorbed by the small intestine providing a multitude of health benefits. The 18-carbon polyunsaturated fatty acids (PUFAs), making up about 2.3% of the FAs in milk, have been linked to improving mental health, reducing inflammation, inhibiting some cancers, and preventing many chronic illnesses such as cardiovascular disease, diabetes, and obesity [1,2,3,4,5]. The major ‘healthy’ FAs are the omega-3 FAs and conjugated linoleic acids (CLA). The primary omega-3 FA in milk is α-linolenic acid (*cis*-9, *cis*-12, *cis*-15 18:3). The CLAs are isomers of linoleic acid (*cis*-9, *cis*-12 18:2) with rumenic acid (*cis*-9, *trans*-11 18:2), one of the most bioactive of the CLAs, comprising 92% of the total CLAs in milk [3].

Overall, milk is considered to be a highly digestible food. Yet there are many processing factors, such as homogenization and high heat treatments, which are known to alter the stability and structure of components in milk [6] that may influence digestibility [7]. Homogenization at pressures of 10–25 MPa ruptures the protective milk fat globular membrane (MFGM) from the fat droplet and reduces it to smaller droplets (1–3 μm) [8]. High heat treatments are used to eliminate pathogens and reduce spoilage bacteria in order to extend the shelf life of milk. Common methods include high temperature, short time (HTST) pasteurization at 72 °C for 15 s or ultra-high temperature (UHT) pasteurization at 135 °C for 2 s.

Although attempts to study the effects of processing on milkfat digestion have been made, it is difficult to compare results because of the variety of methods employed. One in vivo study used fasting rats fed raw, pasteurized, or pasteurized/homogenized cream. Results showed that pasteurized cream under gastric conditions had the lowest levels of long-chain (≥14-carbons) FAs. Pasteurized/homogenized cream under intestinal conditions had the lowest levels of medium- and long-chain (≥8-carbons) FAs [9]. Another approach used human gastric juices containing pepsin and lipases as well as porcine pancreatin and bile salts in the in vitro digestion of milk samples with different-sized lipid droplets. Results showed that the small native lipid droplets released more FAs under gastric and intestinal conditions than larger native or smaller homogenized droplets. The size of the droplet also influenced the FAs released, which suggested that the size of the droplet may alter the positioning of the FAs at the droplet surface (enzyme access interface) [10].

Previous research in our laboratory used yet another approach based on the simulated fasting human gastro-intestinal model, which uses porcine pepsin and pancreatin and bovine bile extract [11] to study the digestibility of raw and processed milk [12]. Although free fatty acids (FFAs) were not released during gastric digestion, raw and homogenized samples released more FFAs than the pasteurized samples during intestinal digestion. However, the rate of FA release began to slow 30 min after lipase addition, and flattened after 90 min because of product inhibition. Comparison of FA profiles prior to digestion and after 3 h of gastro-intestinal digestion showed that stearic acid (18:0) and oleic acid (18:1) were released faster than the other FAs. All processing treatments showed similar trends in digestion for the same FA, but linoleic acid (*cis*-9, *cis*-12 18:2), its CLA isomers, and α-linolenic acid (*cis*-9, *cis*-12, *cis*-15 18:3) were not reported. Similar digestion trends were found in a follow up study that compared the quantity of FFAs released during in vitro gastro-intestinal digestion of raw and processed milk from grazing organic and confined conventional cows [13]. In that study, over half of the FFAs released in the intestinal phase were generated in the first 15 min after lipase addition. Milk from confined conventional herds, which had higher levels of palmitic (16:0) and stearic (18:0) acids, released more FFAs compared to the grazing organic herd. These FAs are typically located on the sn-1 and sn-3 TAG positions that are preferred by pancreatic lipase.

The diversity of the approaches used to study the digestion of milk offers unique opportunities to understand different aspects of the digestion process. It is imperative that the FA profiles include the healthy C18 FAs, not only to provide baselines for future research, but to aid in understanding the release of bioactive FAs in dairy foods. This work, stemming from our organic milk study [13], (1) provides complete and accurate FA profiles for milk and digested samples using a more sensitive column and (2) uses the FA profiles to evaluate the effect of homogenization and/or heat pasteurization on the gastro-intestinal digestion of milkfat.

## 2. Materials and Methods

### 2.1. Processing of Milk

Three shipments of raw milk (38 L) were obtained from an actively grazing certified organic herd located in Berks County, PA between mid-July and early September and processed that afternoon as described by Van Hekken et al. [13] in our dairy pilot plant facilities. Briefly, milk was standardized to 3.25% fat and divided into five portions. One portion remained unheated raw (R) milk control. Another portion was high temperature, short time (HTST) pasteurized (P) at 72 °C for 15 s using a Universal Pilot Plant (UPP, Waukesha Cherry-Burrell, Philadelphia, PA, USA). A third portion was HTST pasteurized and homogenized (HP) using the two-stage homogenizer (17.2/10.3 MPa) attached to the UPP. Another portion of the raw milk was warmed to 60 °C to liquefy the fat before being homogenized (H) in the off-line UPP homogenizer. The last portion was ultra-high temperature pasteurized at 135 °C for 2 s using a FT 74 P/T HTST/UHT Processing System (Armfield, Denison, IA, USA) and homogenized (HU) in the off-line UPP homogenizer. Milk samples were stored at 4 °C until digested and aliquots of milk for FA analysis were frozen at −35 °C until analyzed.

### 2.2. Digestion of Milk

Digestion of milk was based on the in vitro human fasting model by Gallier et al. [14] as modified by Tunick et al. [12] and described by Van Hekken et al. [13]. Raw milk samples were digested the next day and all treatments were digested within 3 days after processing; digestions were conducted once. Briefly, 50 mL of warmed (37 °C) simulated gastric fluid [11] consisting of 0.2% NaCl and 0.32% pepsin (P7000 from porcine gastric mucosa, Sigma-Aldrich, St. Louis, MO, USA), pH 1.2, was added to 100 mL of warmed (37 °C) milk, and adjusted to pH 1.5 with 6 M HCl (Fisher Scientific, Fair Lawn, NJ, USA). The mixture was constantly shaken in a water bath at 37 °C for 60 min to produce a gastric digest. A mixture of 1:1 gastric digest: simulated intestinal fluid (SIF) [11] containing 0.68% KPO_4_ (Fisher) was adjusted to pH 7.0 with 0.2 N NaOH (Fisher). Bile salts (B8631 porcine bile extract, Sigma-Aldrich, St. Louis, MO, USA) were added to a concentration of 5 mg/mL SIF, along with porcine pancreatin (P1750, Sigma-Aldrich) to a concentration of 1.6 mg/mL SIF. The pH was not adjusted during digestion. Aliquots of the intestinal digests were taken after 120 min and centrifuged at 5000× *g* for 30 min at 10 °C (Avanti J-301; Beckman Coulter, Inc., Brea, CA, USA) to obtain the lipid portion, which was immediately frozen at −35 °C (REVCO ^TM^ DxF; Thermo Scientific, Inc., Waltham, MA, USA) until analyzed.

### 2.3. Fatty Acid Profiles

Profiles of the FAs still bound to the TAG in each processed milk and digested samples were obtained based on a modified version of the procedure described by Tunick et al. [12]. Briefly, the FAs were converted to methyl esters (FAME) by reacting with sodium methoxide (Sigma-Aldrich) (free FAs were not detected by this method) and 0.5 μL samples were injected into a Trace 1300 gas chromatograph equipped with a flame ionization detector set at 270 °C (Thermo Scientific, Waltham, MA, USA) and a CP-Sil 88 column (100 m × 0.25 mm; Agilent Technologies, Wilmington, DE, USA). Helium flow was 1.0 mL/min and the injector was 20 mL/min split flow with the inlet temperature at 250 °C. The initial oven temperature was 80 °C and was increased at 4 °C/min to 220 °C and held for 5 min; the oven temperature was then increased at 4 °C/min to 240 °C and held for the remaining 10 min: The instrument’s software was used to calculate percentages of FA. The internal standard added before esterification was glyceryl trinonanoate (Sigma-Aldrich), and reference standards consisted of 4–24-carbon methyl esters and conjugated methyl linoleate (Nu-Chek-Prep, Elysian, MN, USA). Data collection started with caprylic acid (8:0).

### 2.4. Statistical Analysis

FA profile means for each shipment were used to determine the effect of milk processing, digestion, and the processing-digestion interaction on the FAs present in the samples. Statistical software was used to conduct analysis of variance (ANOVA) using the PROC MIXED statements and the Bonferroni LSD test to determine significant differences (*p* < 0.05) (Statistical Analysis System (SAS) for Windows, version 9.4; SAS Institute Inc., Cary, NC, USA). The repeatability of the digestion method was determined using the PROC GLM (SAS) to obtain the coefficient of variance (CV) and standard deviation (SD) for the undigested (*n* = 15) and digested milk (*n* = 11) samples.

## 3. Results and Discussion

### 3.1. GC System

Use of the longer (100 m) fused silica column coated with the highly polar cyanoalkyl polysiloxane stationary phase, CP Sil 88, resulted in finer resolution of peaks and identification of more monounsaturated FAs (MUFAs) and odd-numbered FAs. In total, 8 saturated FAs (SFA), 6 MUFAs, and 3 of the healthy PUFAs (Table 1) were quantified, which is an improvement over our previous GC system that used a 60 m fused silica column with a stationary phase of biscyanopropyl/cyanopropylphenyl siloxane [12]. The major CLA in milk, rumenic acid (*cis*-9, *trans*-11 18:2), was quantified, whereas the other CLA isomers were not detected. Although 400 different FAs that have been identified in milk, only 20 FAs make up 90% of the FAs found in milk [15]. The FAs present in trace amounts, such as the other CLAs, will be difficult to isolate and identify. Further modification of the FA analysis protocol, both in sample preparation and the GC method program, may resolve overlapping peaks to identify even more FAs, including other CLAs and omega-3 FAs.

### 3.2. FA Profiles

Table 1 shows the types and levels of FAs in the milk and digested samples. The FA profiles of the milk prior to digestion were similar to those of previously published studies of milk from actively grazing cows in the USA [16,17,18]. All of the profiles were dominated by palmitic (16:0) and oleic (18:1) acids, comprising over 50% of the FAs. The myristic (14:0) and stearic (18:0) acids comprised about 20% of the FAs. Rumenic acid (*cis*-9, *trans*-11 18:2) was present at nearly 1%, and none of the other 20 isomers reported in the milk [19] were detected. Statistical analysis of the milk and digested milk data resulted in coefficient of variance of 10.9 ± 0.44 and 13.6 ± 0.52, respectively, for the overall repeatability of the experiment.

The digestion system we used did not add lipase until the intestinal phase [14], and we did not detected any FFAs in the digest until then. Other studies that used the in vivo rat model [9] or human gastric juices in an in vitro model [10] reported the release of FFAs during the gastric phase. It has been suggested that gastric lipolysis enhances the efficiency of pancreatic lipase [4,10].

The profiles changed markedly with simulated intestinal digestion, due to the lipolytic activity of the added pancreatic lipases supported by the bile salts. The concentrations of many of the 18-carbon FAs decreased significantly (*p* < 0.05), which indicated the release of FFAs into the digest (Table 1). The amounts of myristic (14:0) and palmitic (16:0) acids in the digested samples appeared to increase (Table 1), although they were being released but at a slower rate than the other FAs. The slower release rate resulted in their higher concentration in the recovered digested fat sample. In bovine milk, 45–65% of myristic (14:0) and palmitic (16:0) acids are located in the sn-2 (center) position of the triacylglyceride molecule [20], partially protecting it from lipolysis. In contrast, the 18-carbon FAs (18:0, 18:1, 18:2 and 18:3) are situated in the sn-1 and sn-3 (outer) positions [20], allowing them to be readily released.

The reductions in 18-carbon FAs may be more easily visualized by noting their ratios to myristic acid before and after digestion (Table 2). The ratios for 18:1, 18:2 and 18:3 substantially decreased (*p* < 0.05) with digestion. Homogenized heat treated (HP and HU) samples tended to have lower ratios for oleic (18:1), linoleic (*cis*-9, *cis*-12 18:2), rumenic (*cis*-9, *cis*-11 18:2), and α-linolenic (*cis*-9, *cis*-12, *cis*-15 18:3) acids. These levels were reduced by more than half when the HP and HU samples were digested, and their ratios to myristic acid (14:0) were significantly lower than the corresponding ratios of the raw, homogenized and pasteurized samples. Homogenization or HTST pasteurization alone did not produce these effects, suggesting that the combination of two treatments may increase digestibility of these four FAs and will require further bench and clinical research to elucidate.

Overall, our results are similar to findings from other digestion studies [9,12]. The in vivo rat study also reported that pasteurized/homogenized cream released the most FAs containing ten or more carbons, but only reported the 18-carbon FAs stearic (18:0), oleic (18:1), and linoleic (*cis*-9, *cis*-12 18:2) acids [9]. An earlier in vitro study from our lab reported greater release of stearic (18:0) and oleic (18:1) acids from homogenized and heat processed milk [12], but did not report results for the PUFAs.

Unlike our study, in vitro studies using human enzymes showed higher levels of digestion from smaller fat droplets, but found homogenization and UHT heat treatment did not enhance milk digestion [10]. They reported that the size of the lipid droplet and its surface (covered with the MFGM or casein/whey proteins) were key to lipid digestion. They also suggested that pancreatic lipases had a lower affinity to the casein coating that forms around the smaller homogenized fat droplets, thereby limiting the release of FAs.

## 4. Conclusions

Findings from this study showed that the use of the longer, highly polar GC column improved the resolution, identification, and quantification of the FAs present in milk and the in vitro digested milk samples. Release of the FAs was easily tracked by comparing the FA profiles before and after digestion. The 18-carbon FAs were rapidly released during intestinal digestion, although care must be taken when interpreting all FA release results to take into account the distribution of the FAs in the diminishing quantity of fat at the end of digestion. While our results suggest that pasteurizing and homogenizing milk may lead to better digestibility of healthy FAs, more research is needed using animal and human models to further investigate this phenomenon.

The diversity of the systems that have been used to digest milk offers unique opportunities to understand certain aspects of the digestion process, which, when combined, will improve our understanding of the effects that processing may have on the human health benefits from consuming milk-based foods.

## Figures and Tables

**Table 1:** Fatty acid concentrations (g/100 g) for differently processed milk before and after a 3 h gastro-intestinal digestion.

Fatty Acid	Common Name	R	H	P	HP	HU	SE
Undigested milk							
8:0	Caprylic acid	1.20 ^a^	1.38 ^a^	1.17 ^a^	1.58 ^a^	1.56 ^a^	0.201
10:0	Capric acid	2.95 ^abc^	3.57 ^ab^	3.07 ^abc^	2.63 ^abc^	3.72 ^a^	0.289
12:0	Lauric acid	3.39 ^a^	4.15 ^a^	3.67 ^a^	3.67 ^a^	4.32 ^a^	0.274
14:0	Myristic acid	11.25 ^c^	13.35 ^c^	11.89 ^c^	12.28 ^c^	12.57 ^c^	0.477
14:1	Myristoleic acid	1.22 ^a^	1.52 ^a^	1.13 ^a^	1.30 ^a^	1.07 ^a^	0.352
15:0		1.19 ^a^	1.42 ^a^	1.32 ^a^	1.31 ^a^	1.28 ^a^	0.284
15:1		0.19 ^c^	0.21 ^bc^	0.22 ^bc^	0.22 ^bc^	0.24 ^bc^	0.030
16:0	Palmitic acid	29.10 ^b^	30.57 ^b^	29.69 ^b^	28.52 ^b^	28.46 ^b^	1.038
16:1	Palmitoleic acid	1.30 ^a^	1.53 ^a^	2.31 ^a^	1.79 ^a^	2.69 ^a^	0.312
17:0	Margaric acid	0.72 ^a^	0.69 ^a^	0.79 ^a^	0.74 ^a^	0.74 ^a^	0.128
17:1		0.30 ^a^	0.31 ^a^	0.30 ^a^	0.30 ^a^	0.29 ^a^	0.180
18:0	Stearic acid	13.85 ^a^	11.27 ^a^	11.94 ^a^	12.68 ^a^	10.59 ^a^	1.292
*cis*-9 18:1	Oleic acid	23.89 ^a^	21.14 ^a^	23.54 ^a^	24.15 ^a^	24.23 ^a^	1.034
*trans*-11 18:1	Vaccenic acid	3.85 ^a^	3.39 ^ab^	3.50 ^ab^	3.65 ^ab^	3.47 ^ab^	0.172
*cis*-9, *cis*-12 18:2	Linoleic acid	3.74 ^a^	3.72 ^a^	3.64 ^a^	3.52 ^a^	3.36 ^a^	0.203
*cis*-9, *trans*-11 18:2	Rumenic acid	0.97 ^a^	0.97 ^a^	0.93 ^ab^	0.92 ^ab^	0.81 ^ab^	0.056
*cis*-9, *cis*-12, *cis*-15 18:3	α-Linolenic acid	0.77 ^a^	0.78 ^a^	0.67 ^ab^	0.69 ^ab^	0.63 ^abc^	0.044
Digested milk							
8:0	Caprylic acid	1.47 ^a^	0.92 ^a^	0.98 ^a^	0.55 ^a^	0.76 ^a^	0.201
10:0	Capric acid	2.16 ^abc^	3.79 ^ab^	2.16 ^a^	1.94 ^bc^	1.50 ^c^	0.289
12:0	Lauric acid	4.24 ^a^	4.64 ^a^	3.67 ^abc^	4.11 ^a^	3.75 ^a^	0.274
14:0	Myristic acid	18.13 ^ab^	17.50 ^b^	17.48 ^a^	20.67 ^a^	19.62 ^ab^	0.477
14:1	Myristoleic acid	2.20 ^a^	2.58 ^a^	2.31 ^b^	2.00 ^a^	2.15 ^a^	0.352
15:0		1.40 ^a^	1.88 ^a^	2.09 ^a^	1.99 ^a^	2.12 ^a^	0.284
15:1		0.22 ^bc^	0.22 ^bc^	0.49 ^a^	0.37 ^ab^	0.31 ^abc^	0.030
16:0	Palmitic acid	37.60 ^a^	38.50 ^a^	39.82 ^a^	41.59 ^a^	43.77 ^a^	1.038
16:1	Palmitoleic acid	2.58 ^a^	2.05 ^a^	2.33 ^a^	2.21 ^a^	1.74 ^a^	0.312
17:0	Margaric acid	1.11 ^a^	1.22 ^a^	0.88 ^a^	0.94 ^a^	1.00 ^a^	0.128
17:1		0.81 ^a^	0.33 ^a^	0.45 ^a^	0.75 ^a^	0.81 ^a^	0.180
18:0	Stearic acid	7.59 ^a^	6.97 ^a^	8.06 ^a^	7.76 ^a^	8.19 ^a^	1.292
*cis*-9 18:1	Oleic acid	13.21 ^b^	13.47 ^b^	12.54 ^b^	9.70 ^b^	8.82 ^b^	1.034
*trans-11* 18:1	Vaccenic acid	3.36 ^abc^	2.22 ^c^	2.86 ^abc^	2.82 ^bc^	2.90 ^abc^	0.172
*cis*-9, *cis*-12 18:2	Linoleic acid	2.89 ^ab^	2.76 ^ab^	2.81 ^ab^	1.99 ^b^	1.97 ^b^	0.203
*cis*-9, *trans*-11 18:2	Rumenic acid	0.60 ^bc^	0.59 ^bc^	0.57 ^bc^	0.33 ^c^	0.33 ^c^	0.056
*cis*-9, *cis*-12, *cis*-15 18:3	α-Linolenic acid	0.42^bcd^	0.38 ^cd^	0.41 ^bcd^	0.28 ^d^	0.27 ^d^	0.044

Processing treatments included: raw, R; homogenized, H; pasteurized, P; homogenized and pasteurized, PU; and homogenized and UHT pasteurized, HU. ^a–d^ Means for the same fatty acid not sharing the same letter are significantly different (*p* < 0.05). Standard error, SE.

**Table 2:** Ratios of 18-carbon fatty acids to myristic acid (14:0) for differently processed milk before and after a 3 h gastro-intestinal digestion.

Fatty Acid	Common Name	R	H	P	HP	HU	SE
Undigested milk							
18:0	Stearic acid	1.23 ^a^	0.85 ^ab^	1.02 ^ab^	1.03 ^ab^	0.85 ^ab^	0.116
*trans-11* 18:1	Vaccenic acid	0.34 ^a^	0.26 ^abc^	0.30 ^a^	0.30 ^a^	0.28 ^ab^	0.015
*cis-9* 18:1	Oleic acid	2.12 ^a^	1.59 ^a^	1.98 ^a^	1.98 ^a^	1.93 ^a^	0.104
*cis-9, cis-12* 18:2	Linoleic acid	0.33 ^a^	0.28 ^ab^	0.31 ^a^	0.29 ^a^	0.27 ^abc^	0.018
*cis-9, trans-11* 18:2	Rumenic acid	0.086 ^a^	0.073 ^a^	0.078 ^a^	0.075 ^a^	0.065^ab^	0.005
*cis-9, cis-12, cis-15* 18:3	α-Linolenic acid	0.069 ^a^	0.058 ^a^	0.056 ^a^	0.056 ^a^	0.050 ^a^	0.003
Digested milk							
18:0	Stearic acid	0.42 ^b^	0.40 ^b^	0.47 ^ab^	0.38 ^b^	0.42 ^b^	0.116
*trans-11* 18:1	Vaccenic acid	0.19 ^bcd^	0.13 ^d^	0.16 ^cd^	0.14 ^d^	0.15 ^d^	0.015
*cis-9* 18:1	Oleic acid	0.73 ^b^	0.77 ^b^	0.72 ^b^	0.47 ^b^	0.44 ^b^	0.104
*cis-9, cis-12* 18:2	Linoleic acid	0.16 ^bcd^	0.16 ^cd^	0.16 ^bcd^	0.10 ^d^	0.10 ^d^	0.018
*cis-9, trans-11* 18:2	Rumenic acid	0.034^bc^	0.035^bc^	0.034^bc^	0.016 ^c^	0.017 ^c^	0.005
*cis-9, cis-12, cis-15* 18:3	α-Linolenic acid	0.024 ^b^	0.021 ^b^	0.024 ^b^	0.013 ^b^	0.014 ^b^	0.003

Processing treatments included: raw, R; homogenized, H; pasteurized, P; homogenized and pasteurized, PU; and homogenized and UHT pasteurized, HU. ^a–d^ Means for the same fatty acid not sharing the same letter are significantly different (*p* < 0.05). Standard error, SE.
